# Beyond AQP-4: convergent glymphatic-meningeal lymphatic dysfunction underlying multifactorial migraine pathogenesis

**DOI:** 10.3389/fimmu.2026.1875765

**Published:** 2026-06-26

**Authors:** Meng-fan Yang, Mao-mei Song, Ying-jie Gao, Ting-yan Chen, Sui-yi Xu

**Affiliations:** 1 Department of Neurology, Headache Center, Shenzhen Baoan People’s Hospital, The Second Affiliated Hospital of Shenzhen University, Shenzhen, Guangdong, China; 2 Department of Neurology, Headache Center, The First Hospital of Shanxi Medical University, Taiyuan, Shanxi, China

**Keywords:** CGRP, CSD, glymphatic system, immune surveillance, meningeal lymphatics vessel, neuroimmunology, neuroinflammation, perivascular space

## Abstract

The glymphatic system (GS) functions as a critical pathway for waste clearance from the brain, facilitating soluble protein and metabolite drainage. Recently, GS dysfunction has emerged as a potential contributor to migraine pathophysiology. GS operates similarly to the peripheral lymphatic system, dependent on astrocytes for metabolic waste removal. The clearance process involves cerebrospinal fluid entering the peri-arterial spaces, moving into the interstitial fluid via aquaporin-4 (AQP-4) channels at astrocyte feet, and eventually being drained into the cervical lymph nodes. As a downstream effector of the glymphatic system (GS), meningeal lymphatic vessels (MLVs) play a critical role in immune surveillance and regulation of cerebrospinal fluid (CSF) efflux. Calcitonin gene-related peptide (CGRP) is primarily involved in pain transmission and neuroinflammation within the nervous system. Within MLVs, CGRP modulates CSF outflow by promoting VE-cadherin rearrangement, thereby influencing pain responses in migraine mice. GS dysfunction has been observed in mice with migraine and may associate with cortical spreading depression (CSD)-induced transient perivascular space (PVS) closure. GS dysfunction has also been observed in the nitroglycerin (NTG)-induced mice migraine model. Consequently, this dysfunction might lead to the accumulation of CGRP, reactive oxygen species, and inflammatory factors, contributing to migraine initiation. In addition, CSD, a key mechanism in migraine aura, is postulated to induce transient PVS closure, disrupting GS flow. Further, impaired GS clearance would potentiate glutamatergic signaling and trigger neuroinflammation. Furthermore, AQP-4, a key component of GS, plays a crucial role in maintaining PVS function and modulating neuroinflammation. Reduced expression and impaired polarization of AQP4 may further impair GS clearance, leading to the accumulation of pathogenic mediators. GS dysfunction might be exacerbated by CSD and neuroinflammation. Further research is warranted to elucidate the underlying mechanisms and explore potential therapeutic targets aimed at restoring GS function in patients with migraine.

## 1 Introduction

In 1914, neurosurgeon Lewis Weed injected a dye into the cerebrospinal fluid (CSF), which rapidly appeared in the cervical lymph nodes, suggesting that CSF drainage occurs not only in the cerebral venous system but also within the lymphatic system (1). The glymphatic system (GS), which is similar to the human lymphatic system, was first described in 2012 (2). GS flow moves in the same direction as blood flow and is propelled forward by arterial wall pulsations (3). Previously, the brain was believed to lack a lymphatic system (4). However, true lymphatic vessels have been demonstrated in the dura mater (5). The meningeal lymphatic system further drains metabolic wastes from the CSF and interstitial fluid (ISF) into the cervical lymph nodes (6, 7), eventually returning to the venous system (8, 9). Meningeal lymphatic vessels (MLVs) are situated around the dural venous sinuses and express canonical lymphatic endothelial cell markers, including vascular endothelial growth factor receptor 3, Prospero homeobox protein 1, podoplanin, lymphatic vessel endothelial hyaluronan receptor 1, and CD31 (10). ISF is the biological fluid located within the intercellular spaces and surrounding capillaries. It comprises water, proteins, solutes, and components of the extracellular matrix, thereby constituting the cellular microenvironment (11). The constituents of ISF are highly dynamic and markedly modulated by regional metabolic processes. The key difference between ISF and CSF lies in the substantially higher protein concentration of ISF, which resembles that of plasma (12). GS acts as the “upstream” of the brain for waste removal and downstream into the true lymphatic system, the meningeal lymphatic system (2, 13).

GS is size-dependent (astrocytes are tightly connected) (14, 15), unidirectionally polarized (inflow and outflow occur via the periarterial and perivenous gaps, respectively) (2), and spatiotemporally regulated (perivascular space (PVS) is region-specific and influenced by circadian rhythms and respiratory system) (16–18). Moreover, as a highly organized fluid transport system, the most central function of GS is waste removal, serving as a substitute for lymphatic vessels and facilitating soluble protein and metabolite drainage (2, 19). It has been demonstrated that beta-amyloid (Aβ) is cleared via the GS in rodent eyes (20). Aquaporin-4 (AQP-4) serves as the most critical component of the GS in the CNS, representing the most abundant aquaporin in the brain, spinal cord, and optic nerves (21). GS also removes extracellular tau protein and prevents its aggregation (22). AQP-4-deficient mouse models of GS dysfunction revealed impaired Aβ clearance and susceptibility to Aβ plaque formation (23). Further, the GS plays a role in nutrient transport (such as lipid) throughout the brain (24). Finally, a potential immune function exists, as the MLV is a key drainage pathway for the CSF into the periphery and may contribute to the inflammatory response and central nervous system (CNS) immune surveillance (25). However, how inflammatory factors reach the brain parenchyma and trigger an inflammatory response remains unclear (26). This review aimed to explore the roles of the GS and MLVs in the pathophysiology of migraine.

## 2 GS-MLVs function

**Figure 1:**
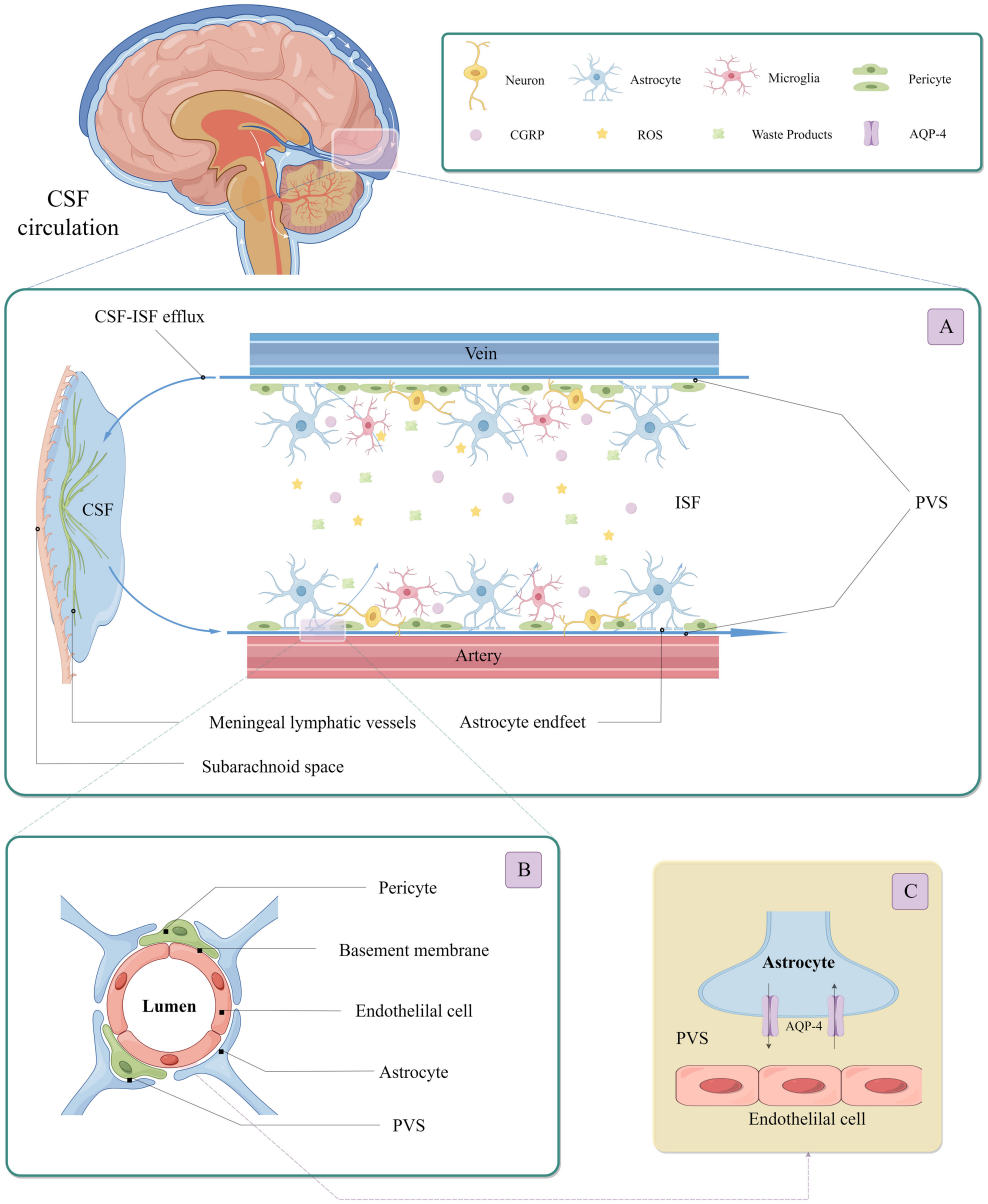
Waste clearance function of the GS operates in parallel with CSF circulation. **(A)** Cerebrospinal fluid **(CSF)** is produced in the subarachnoid space and subsequently enters the periarterial space. Through the driving action of astrocytic aquaporin-4 (AQP-4), CSF then enters the interstitial fluid **(ISF)** space, which serves as the primary site for waste clearance and CSF-ISF mixing. The mixed metabolic waste products (including calcitonin gene-related peptide [CGRP] and reactive oxygen species [ROS]) subsequently drain through the perivenous space into meningeal lymphatic vessels, ultimately reaching peripheral circulation via cervical lymph nodes. Both periarterial and perivenous spaces are collectively termed the perivascular space (PVS). **(B)** PVS is composed of the vascular wall, basement membrane, and AQP-4-enriched astrocytic endfeet. **(C)** AQP-4 facilitates the transport of CSF and metabolic waste products within the GS and plays a critical role in regulating the PVS and maintaining GS function.

The function of the GS is similar to that of the peripheral lymphatic system in that both are astrocyte-dependent. Metabolic waste removal by the GS can be broadly categorized into three processes (2, 13, 26) (1): CSF originating in the subarachnoid space enters the peri-arterial spaces in a bulk-flow driven manner (2); AQP-4 at the end of the astrocyte foot promotes CSF movement from the peri-arterial compartment into the ISF space, enabling CSF-ISF mixing and metabolic waste removal (27); ISF, waste products, and CSF-ISF mixtures are driven into the perivenous space and eventually drained into the MLVs and cervical lymph nodes for clearance and breakdown (**Figure 1**). Metabolic waste removal in the GS is closely related to PVS, which is defined as the space around the cerebral vessels that corresponds to the sum of the para-arterial and para-venous spaces. The inner wall of the PVS is the vascular wall, whereas the outer wall is composed of the basement membrane and AQP-4 at the end of the astrocytic foot (28, 29). AQP-4 may promote CSF-ISF exchange (30, 31). PVS, also known as the “prelymphatic system, “ is an extension of the subarachnoid space, which is filled with CSF and serves as a site for convective CSF exchange with ISF (32). PVS also serves as an outlet for metabolites produced by the GS after ISF clearance [including excitatory and inflammatory substances produced after cortical spreading depression (CSD)] (33). Initial lymphatic vessels are composed of discontinuous lymphatic endothelial cells (34, 35), allowing interstitial fluid, macromolecules, soluble antigens, and immune cells (including antigen-presenting cells) to enter (36). The MLVs are capable of clearing CSF, metabolic byproducts, aged cells, and immune cells from the brain parenchyma (37). Moreover, the MLVs play an immune surveillance role in the central nervous system (CNS), promoting the migration of immune cells to the deep cervical lymph nodes (DCLN) and regulating peripheral immunity (36, 38). Finally, all metabolic wastes are transported to the cervical lymph nodes. Thus, GS and PVS are inextricably linked both structurally and functionally. Moreover, both are indispensable and share the burden of CNS waste removal.

CSF transport is initiated during non-rapid eye movement periods (8). Therefore, GS flow is closely related to sleep. GS is more active during sleep and anesthesia (17). This may help explain why sleep alleviates migraine attacks (39). The clearance of harmful metabolites such as Aβ by the mouse brain during sleep was equivalent to twice the rate during wakefulness (17). Further, GS function is influenced by various factors, such as body posture, stress, and adrenergic tension may also regulate lymphatic system activity (40, 41). Consequently, GS efficiency was higher in lateral and supine rats than in prone rats, consistent with the natural resting and sleeping postures (40). Aging is believed to be an important regulator of the brain’s lymphatic system (42). Conversely, GS dysfunction also affects aging, possibly through mechanisms such as chronic inflammation, mitochondrial and oxidative stress, and protein homeostasis (43).

## 3 GS and migraine: clinical findings

Currently, no ideal technique is available for studying human GS despite the evolving technology of functional lymphatic imaging. Various techniques are available for providing different information on human GS function, including magnetic resonance, positron emission tomography, and ultrasound (44, 45). Magnetic resonance sequences, including three-dimensional (3D) FLAIR, coronal T2-weighted, 3D T1-weighted imaging, and diffusion tensor imaging (DTI), are available for the GS and PVS study (46). Intravenous gadolinium contrast agent enhancement combined with magnetic resonance imaging is a more non-invasive and commonly used study method than intrathecal gadolinium-based contrast agents (47). Another non-invasive method not requiring an intravenous or intrathecal injection of gadolinium-based contrast agent is DTI analysis along the PVS (DTI-ALPS) (48). The DTI-ALPS index was used to express GS activity, with higher indexes indicating higher GS activity (49).

GS dysfunction by PVS closure in migraine with CSD was first described in a basic study (33). However, a clinical study has presented a different perspective. The study proposed that patients with migraine with and without aura are free of GS dysfunction (50). Furthermore, it has been argued that the above studies did not investigate whether GS dysfunction occurs during migraine chronicity; therefore, a cross-sectional study incorporating both episodic and chronic migraine was conducted (51). All investigations were conducted during the attack-free period of patients with migraine. Notably, the DTI-ALPS index was significantly higher in patients with chronic migraine (CM) than in those with episodic migraine (EM) and healthy controls, suggesting increased rather than impaired GS activity. It is hypothesized that the increased GS function in CM patients may be mediated by downstream vascular responses to CGRP release. Compared with healthy participants, patients with migraine had enlarged PVS (EPVS), which indirectly responded to GS dysfunction (52). The presence of visible EPVS on magnetic resonance imaging (MRI) may indicate GS dysfunction (28, 53). Although most studies have shown that an increased prevalence of EPVS in migraine patients (54, 55), some have reached the opposite conclusion. A population-based Hunt-MRI indicated that no increased dilated PVS in headache patients (56). More recently, a 2024 clinical study with a large cohort showed that DTI-ALPS index was significantly reduced in CM patients compared to both healthy controls and the EM patients (57). It indicates impaired glymphatic function in CM. However, DTI-ALPS tends to be biased owing to the presence of crossed fibers in 90% of the brain’s white matter (58, 59). Recently, Diffusion Kurtosis Imaging ALPS (DKI-ALPS) was employed to assess GS function in patients with migraine during the attack-free period (58). The DKI-ALPS index was higher in patients with migraine than in healthy participants, confirming enhanced GS activity among patients with migraine. A recent study demonstrated that impaired GS function contributes to a higher frequency of attacks in patients with EM. Reduced DTI-ALPS index may serve as a potential non-invasive indicator of GS impairment in patients with high-frequency EM (60). Interestingly, MLV dysfunction in EM correlates with headache intensity, and the authors speculated that this might be related to impaired clearance of inflammatory mediators such as CGRP (61). Therefore, whether EPVS are present in migraine patients remains unclear, and no consensus exists on GS activity (increase, decrease or remain unchanged) in this population, requiring further investigation.

Current GS clinical studies have some limitations (51): first, the sample size was small and not representative of the overall population; second, DTI-ALPS scores are usually calculated at the level of the lateral ventricles, which represents only part of the GS function. Several clinical studies have reached inconsistent conclusions regarding GS pathogenesis in migraine; however, this review will focus on some clinical and experimental evidence favoring GS dysfunction (33, 47, 57, 62). Currently, the two primary animal models employed in migraine mechanism research are as follows (63): CSD model, which elucidates the neurophysiological basis of migraine with aura (64), and the nitroglycerin- induced model, which mimics migraine without aura or chronic migraine (65). In animal studies, both experimental models have revealed GS dysfunction—manifested as transient PVS closure in the CSD model and impaired AQP-4–dependent clearance in the NTG model.

## 4 Meningeal lymphatic vessels

### 4.1 CNS immune surveillance

MLVs are not merely drainage conduits of the central nervous system. Under homeostatic conditions, the meningeal spaces—particularly the dura mater—host a diverse array of immune cells, including T cells (CD4^+^, CD8^+^, and T cell receptor γδ), macrophages, dendritic cells (type 1 and type 2 classical dendritic cells, plasmacytoid dendritic cells, and migratory dendritic cells), natural killer (NK) cells, mast cells, neutrophils, and B cells (both immature and mature). These cells constitute an essential component of meningeal immunity (10, 36, 66). CSF derived from the subarachnoid space drains into the dural compartment. Within the dura, antigens from the brain and CSF are captured and processed by resident antigen-presenting cells (APCs), which subsequently present them to patrolling T cells, thus promoting CNS immune surveillance. T cells egress into the DCLN in a CCR7-dependent manner, a process analogous to inflammation-induced migration of peripheral T cells (67). Both CD4^+^ T lymphocytes and TCRγδ cells are capable of modulating the homeostatic state of CNS neurons and glia through the release of cytokines such as IL-4, IL-17, and IFN-γ (36, 68, 69). Regarding migration, B cells are capable of egressing through MLVs to DCLN in a manner similar to that of T cells. During the exchange of CSF and ISF within the GS, astrocytes and macrophages interact with each other, and APCs along with CNS-derived antigens move from the GS into the CSF (70). Meningeal dendritic cells can enter the CSF-filled spaces and surveil substances crossing the blood-cerebrospinal fluid barrier or BBB (38), thereby enabling the detection of various insults and infections (71). Thus, MLVs and the GS constitute key structural components of CNS immune surveillance. Through the GS-MLV axis, immune cells can egress from the brain parenchyma into the CSF and ultimately reach the peripheral immune system (10, 72).

### 4.2 MLV-CGRP signals regulate the CSF efflux

MLVs serve as a downstream pathway for the drainage of GS metabolites, CNS-derived antigens, and the egress of immune cells (36). In addition to its role in trigeminovascular pain transmission, CGRP targets MLVs endothelial cells, where it promotes VE-cadherin reorganization and tightens endothelial junctions, thereby decreasing MLVs permeability. Consequently, CSF drainage to DCLN is markedly reduced, potentially leading to CSF buildup or dural entrapment of pro-migraine vascular and immune mediators (73, 74). The CGRP receptor is a heterodimer formed by calcitonin receptor-like receptor (gene: *Calcrl*; protein: CLR) and receptor activity-modifying protein 1 (gene: *Ramp1*; protein: RAMP1) (65). The expression level of the CGRP receptor is higher in lymphatic endothelial cells (LECs) than in vascular endothelial cells (75, 76). CLR activation triggers a rapid and robust remodeling of junctional and gap proteins in LECs, thereby tightening the intercellular barrier and reducing lymphatic permeability (77). As an endothelial adhesion molecule, MADCAM1 binds to the α4/β7 integrin (LPAM1^+^) present on immune cells, facilitating immune cell-endothelial cell adhesion (78). Studies in a NTG-induced chronic migraine (CM) mouse model have revealed that meningeal LECs, as well as cultured human LECs exposed to CGRP, exhibit changes in both gene and protein expression. These findings suggest the presence of lymphatic-vascular immune interactions during the CM pathophysiology (65). NTG-induced CGRP signaling activates MLVs capillary termini and upregulates the expression of MADCAM1 and pentraxin-3. These changes may promote the egress of LPAM1^+^ CD4^+^ T cells into DCLN (73). Thus, in the development and progression of migraine, CGRP may not only function as a pain mediator but also influence CSF efflux and neuroimmune interactions through remodeling of the MLVs endothelium.

## 5 Potential role of GS in migraine

Although most studies of GS dysfunction in migraine are based on CSD (33, 62), it has recently been demonstrated that GS dysfunction increases CGRP levels by and exacerbates migraine in a mice model of nitroglycerin-induced migraine (47). CGRP is a neuropeptide containing 37 amino acids that play key roles in migraine development (79). In the NTG-induced migraine mice model, reduced AQP-4 expression, GS dysfunction, and elevated CGRP levels were observed. The authors proposed that CGRP accumulation may result from impaired GS clearance capacity (47); however, this hypothesis lacks direct experimental evidence. Glymphatic drainage blocking results in accumulation of CGRP and reactive oxygen species (ROS) (47, 80). In addition, impaired CGRP and ROS clearance leads to neuroinflammation (81, 82), causing reactive astrocyte proliferation. GS dysfunction manifests in both CSD and NTG migraine models, albeit through distinct mechanisms. In the CSD model, GS dysfunction primarily results from transient closure of PVS (62). Nevertheless, in the NTG model, GS dysfunction arises from AQP-4 downregulation and loss of polarization (47). Collectively, these findings indicate that migraine pathogenesis is intrinsically linked to GS dysfunction across experimental paradigms.

### 5.1 CSD and GS

**Figure 2:**
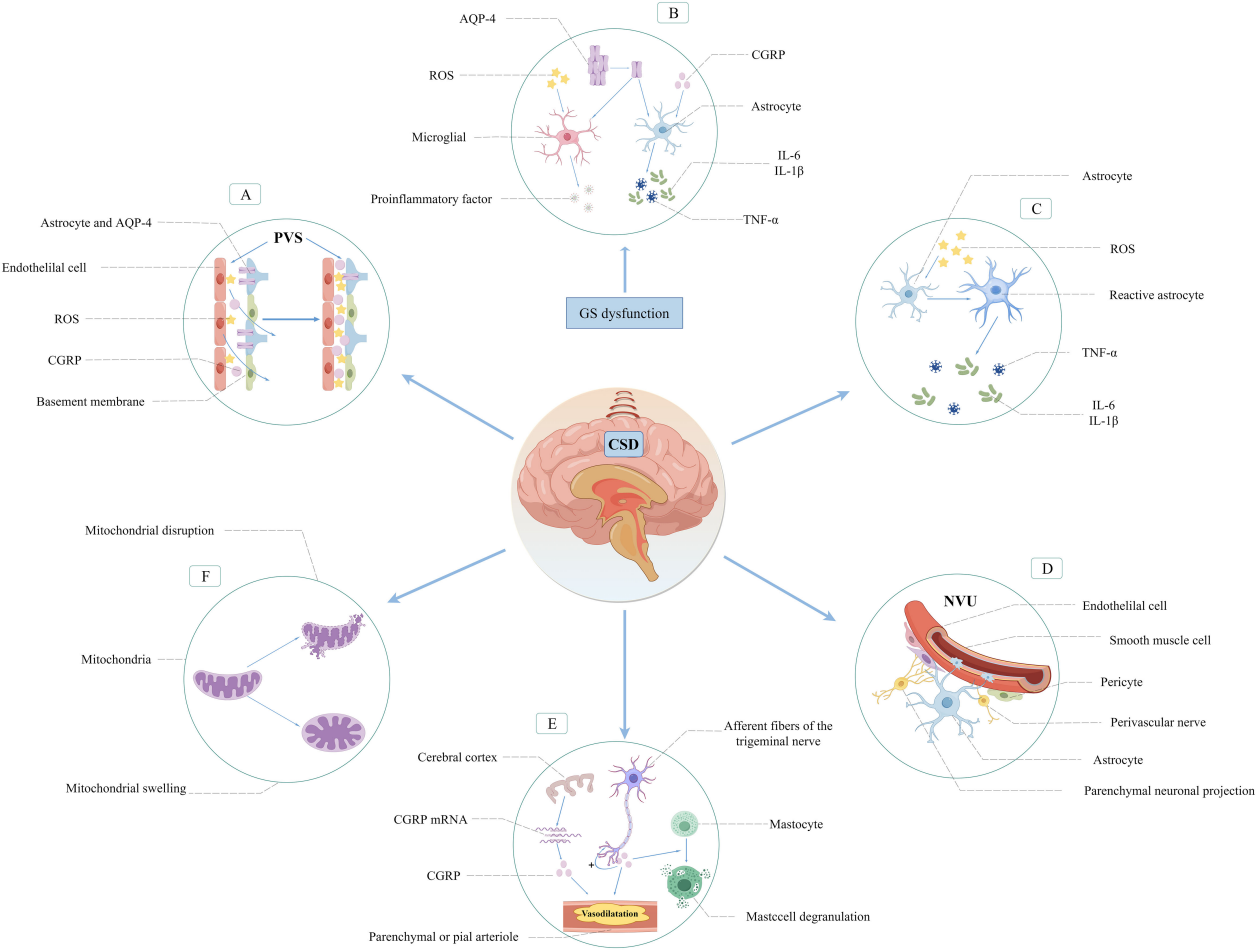
Pathological cascade of glymphatic dysfunction, neuroinflammation, mitochondrial impairment, and neurovascular unit activation. **(A)** Cortical spreading depression **(CSD)** induces narrowing or even transient occlusion of the perivascular space (PVS), leading to substantial accumulation of metabolic waste products within the central nervous system. This exacerbates glymphatic system **(GS)** dysfunction. **(B)** GS dysfunction leads to downregulation of aquaporin-4 (AQP-4) and accumulation of ROS, which subsequently activates microglia and triggers the release of pro-inflammatory cytokines. Concurrently, AQP-4 downregulation and CGRP activate astrocytes, promoting the secretion of inflammatory mediators such as interleukin-6 (IL-6), interleukin-1β (IL-1β), and tumor necrosis factor-α (TNF-α). **(C)** ROS induces hypertrophy and proliferation of normal astrocytes, transforming them into reactive astrocytes. These reactive astrocytes subsequently release increased amounts of inflammatory cytokines, which in turn further activate astrocytes, exacerbating neuroinflammation. **(D)** CSD activates the neurovascular unit (NVU), which comprises astrocytic end feet, parenchymal neuronal projections, perivascular nerves, pericytes, vascular smooth muscle cells, and endothelial cells. **(E)** CSD induces the accumulation of CGRP in both central and peripheral compartments. Peripheral CGRP triggers mastocyte degranulation, promoting neuroinflammation, while simultaneously creating a positive feedback loop that further enhances CGRP release. This self-amplifying pathway ultimately leads to localized vasodilation. **(F)** CSD induces mitochondrial fragmentation and swelling, consequently impairing energy metabolism.

CSD is believed to be a key mechanism in the occurrence of migraine aura (83). CSD spreads slowly across the cortex at a rate of 2–5 mm/min (84), resulting in changes in blood flow and astrocyte endfeet swelling (85, 86). CSD has an extremely high energy demand (87), resulting in an ATP decrease of approximately 50%, which may activate the neurovascular unit (NVU) (**Figure 2**). NVU structural function permitted bi-directional communication between the microvascular system and neurons (88). Thus, GS function in patients with migraine may be affected by vascular dysfunction. Proinflammatory factors and pain mediators (including calcitonin gene-related peptides) after CSD may play an important role in headaches (89, 90). CSD leads to PVS closure because of astrocyte foot swelling (33), which is a major feature during CSD (86). Astrocyte foot is rich in AQP-4, an essential component of PVS and a key structure for GS clearance (91). PVS is an outlet for GS metabolic wastes, and its closure may lead to impaired GS clearance ability (92). Furthermore, inflammatory factor release further might disrupt the PVS (32).

### 5.2 CGRP accumulation and CSD

CGRP is excreted by three routes: venous plasma, CSF, and the lymphatic system (46). CGRP is predominantly expressed in C- and Aδ-type nociceptive nerve fibers (93), but does not readily cross the blood-brain barrier (BBB). Instead, it accesses the CSF via PVS, a process mediated by the GS (94). This mechanism accounts for the fivefold higher concentration of CGRP in CSF compared to plasma (32). Emerging evidence suggests that a subpopulation of CGRP may drain into the superior sagittal sinus through arachnoid granulations, thereby enabling the detection of circulating CGRP in jugular venous blood (92). PVS facilitates CSF-ISF exchange (2, 19). CGRP caused increases in the K^+^-evoked release of aspartate and glutamate (95). Glutamate, the primary excitatory neurotransmitter in the CNS, induces central sensitization through excessive synaptic accumulation, representing a critical mechanism underlying migraine pathogenesis (96). CGRP may further elicit compartmentalized pro-inflammatory mediator releases (81, 82).

A non-synaptic communication pathway between the central and peripheral nervous systems was proposed, in which trigeminal ganglia in all experimental animals directly interface with the CSF. Humans exhibit a similar anatomical arrangement: the ganglion resides within CSF-filled cavities (97). CGRP concentration in trigeminal ganglia doubled following CSD. This suggests that CGRP transported via the CSF to the ganglia may directly contribute to migraine pathogenesis. During CSD, the concurrent elevation of extracellular potassium ions (K^+^) and glutamate coincides with experimentally validated CGRP exocytotic release under K^+^-rich conditions, suggesting a plausible spatiotemporal coupling of CGRP secretion to CSD propagation (98, 99). Atogepant, a selective CGRP receptor antagonist, substantially attenuates the population density of depolarization-active neurons following CSD, mechanistically substantiating the pivotal role of CGRP signaling in propagating CSD-related neuroelectrical dysregulation (64). CSD triggers an acute surge in CGRP release via voltage-gated calcium channel-dependent exocytosis. Furthermore, repetitive CSD episodes induce sustained transcriptional CGRP upregulation (90). CSD events centrally trigger CGRP mRNA release (100), while peripherally activating trigeminal afferent nerves in the meninges induce CGRP secretion, leading to local vasodilation and mastocyte degranulation, creating a positive feedback loop that amplifies further CGRP release (101–103).

Collectively, in migraine pathophysiology, CSD events directly promote CGRP buildup. The bi-directional potentiation between CSD and CGRP exacerbates migraine initiation and progression. Impaired GS clearance might lead to CGRP overaccumulation, but this assumption requires further evidence.

### 5.3 Neuroinflammation and GS

Neuroinflammation occurs in the peripheral and central nervous systems (104), which is primarily characterized by four hallmark features: 1) increased vascular permeability, 2) leukocyte infiltration, 3) glial cell activation, and 4) inflammatory mediator production (104). Neuroinflammation is predominantly mediated by astrocytes, microglia, and the neurovascular unit (105). The accumulation of ROS and inflammatory mediators leads to neuronal degeneration and astrocyte activation (86, 106). Additionally, CSD can trigger an inflammatory response via the opening of neuronal Pannexin 1 megachannels and activation of caspase-1, followed by the activation of astrocytes (103). Following inflammatory injury, astrocytes undergo proliferation and hypertrophy, developing into reactive astrocytes that subsequently release inflammatory mediators (86, 107). These pathological changes collectively exacerbate migraine pathogenesis.

GS plays a critical role in the clearance of ROS and inflammatory mediators. Under conditions of GS dysfunction, excessive ROS in the brain stimulate microglia to produce pro-inflammatory factors (108, 109). Ultimately, these molecules are released into the extracellular space and circulate within the GS (110). Conversely, GS dysfunction may lead to elevated pro-inflammatory cytokines and reduced anti-inflammatory mediators in the brain (80, 111). Impaired glymphatic function affects the brain’s ability to remove inflammatory mediators (112), such as IL-1β, INF-γ, TNF-α, while excessive inflammatory mediators, in turn, exacerbate GS impairment.

CGRP exerts pro-inflammatory effects on cultured astrocytes, suggesting its substantial role in neuroinflammation (113). Firstly, reactive astrocytes may reduce brain clearance and produce cytokines and other inflammatory mediators (114–116). Secondly, a cross-sectional study demonstrated that the reduced function of astrocytic activity can impair the GS (117). Finally, AQP-4 deficiency has been demonstrated to exacerbate nitroglycerin-induced neuroinflammation, amplifying both microglia and astrocyte activation (47), which may lead to increased release of pro-inflammatory mediators, which potentiate nociceptive stimuli and result in nociceptor hyperactivation (118). Furthermore, excessive inflammatory mediators and elevated oxidative stress levels can lead to PVS obstruction, impairing waste clearance in the GS and thereby exacerbating neuroinflammation (119).

### 5.4 AQP-4 and GS

AQP-4 is localized to the perivascular and subpial membranes of astrocytes, where it is expressed as two isoforms: AQP-4M1 and AQP-4M23 (120). The AQP-4M1 isoform moves freely within the plasma membrane and primarily facilitates CSF movement, whereas the AQP-4M23 isoform tends to form orthogonal array particles and restricts astrocyte migration (121, 122). An increased ratio of AQP-4M1 to AQP-4M23 disrupts orthogonal array particles, which play a critical role in AQP-4 polarization (123, 124). The highly polarized distribution of AQP-4 at perivascular astrocytic endfeet constitutes the functional cornerstone of the GS (125, 126). The perivascular localization of AQP-4 in the brain requires the dystrophin-associated complex (DAC) (125, 127). DAC includes α-syntrophin (α-Syn), dystrobrevin, dystrophin, and dystroglycan (23, 125). Loss of α-Syn disrupts the normal polarized distribution of AQP-4 (127, 128), thereby compromising GS function (23, 129). Compared with wild-type mice, mice with targeted disruption of the gene encoding α-Syn exhibit swollen perivascular and subpial astrocytic endfeet in the brain, along with reduced clearance of metabolically derived water (130).

Astrocytic end feet constitute essential structural components of the PVS, and they dynamically regulate PVS dimensions to modulate both the flow velocity and biochemical composition of ISF (62). GS blockade downregulates AQP-4 gene expression and upregulates TNF-α production, thereby exacerbating neuroinflammatory injury (80). AQP-4 facilitates CSF transport GS (131). AQP-4 deficiency impairs 65% of CSF-ISF exchange and reduces Aβ clearance rate by approximately 55% (2), suggesting that the GS is AQP-4-dependent. Notably, astrocyte endfoot swelling following CSD is not mediated by AQP4 but coincides with vascular constriction. However, their experiment did not establish whether CSD directly downregulates AQP4 expression, highlighting the need for further research (62). Sevoflurane enhances Aβ clearance by upregulating AQP-4 expression (132). Aβ is primarily cleared via the GS, demonstrating that AQP-4 serves as a critical component of the GS. Furthermore, in a mouse pain model, AQP-4(−) mice exhibited significantly fewer pain-related behavioral responses compared to AQP-4(+) mice (133).

In a mouse migraine model, reduced AQP-4 expression and diminished perivascular AQP-4 polarization were observed, suggesting that AQP-4 may contribute to GS dysfunction in migraine (47). Nevertheless, it remains unresolved whether GS dysfunction arises from reduced AQP-4 expression or from loss of its polarized distribution. Furthermore, whether defective AQP-4 polarization is caused by abnormalities in cytoskeletal anchoring proteins or simply reflects decreased AQP-4 protein levels is still to be elucidated. It has been demonstrated that in AQP-4-deficient mice, the GS displays impaired clearance of two tracers: mannitol (0.18 kDa) and dextran (500 kDa) (125). In α-Syn knockout mice (Snta1–/–), perivascular AQP-4 is reduced by 90% (130, 134), whereas total AQP-4 expression remains unchanged. This indicates a positive correlation between polarized AQP-4 expression and α-Syn. Compared with wild-type (WT) mice, Snta1–/– mice showed no significant change in mannitol clearance but exhibited reduced clearance of dextran (125). These findings suggest that the pronounced polarized localization of AQP4 alone does not fully explain its role in mediating solute clearance from the brain.

Furthermore, TGN-020 (an AQP-4 inhibitor) increased inflammatory cytokine levels in migraine mice (47), though the causal relationship between reduced AQP-4 expression and migraine remains unclear. A further study revealed that loss of AQP-4 induces GS dysfunction through widespread stagnation of ISF (135). Moreover, AQP-4 plays a critical role in maintaining the balance of neurotransmitters, for example, glutamate and Na^+^ within the synaptic cleft of the CNS (136–138). Consequently, AQP-4 downregulation, impaired glymphatic function, and increased CGRP levels occur concurrently. These findings support an association between impaired neurofluid clearance and CGRP-related migraine signaling. These pathological changes collectively contribute to migraine pathogenesis.

## 6 Conclusions

**Figure 3:**
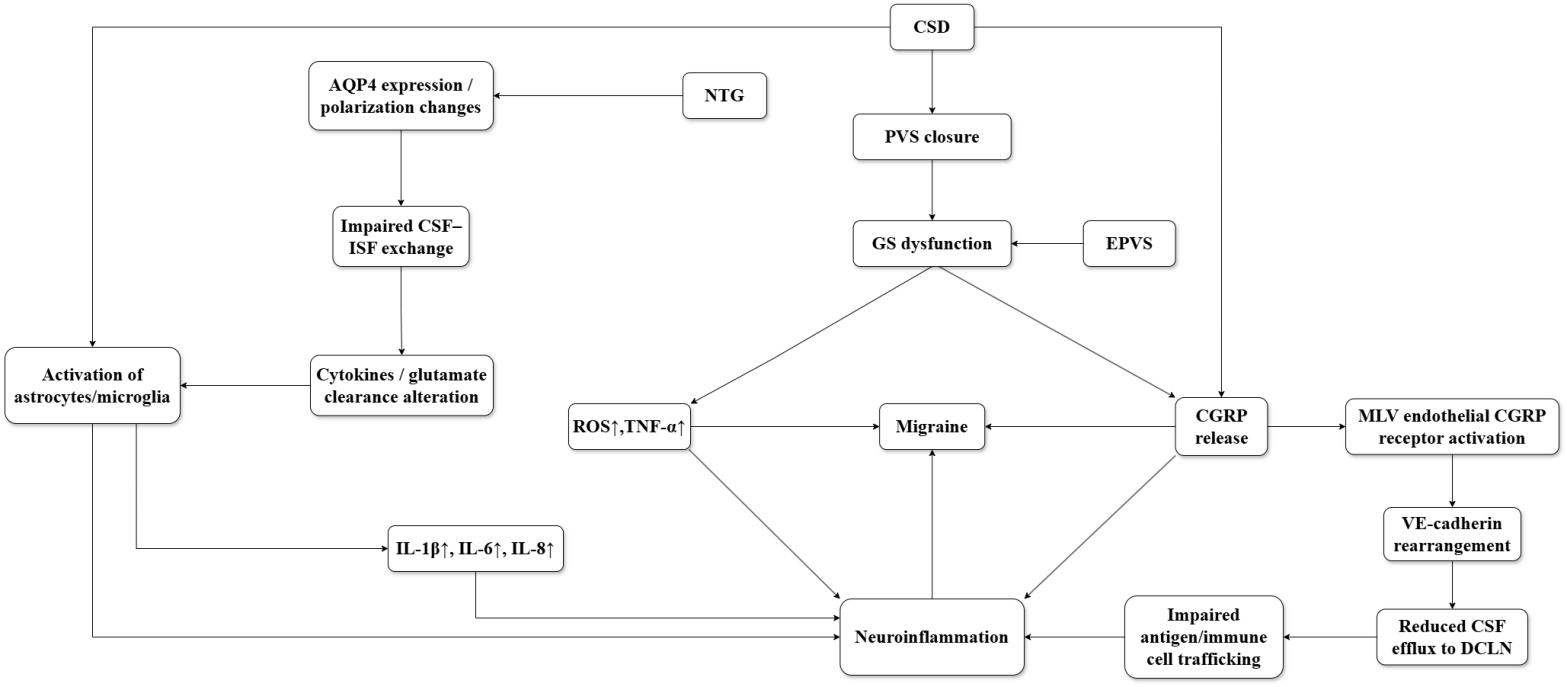
Glymphatic system dysfunction in migraine. CSD, cortical spreading depression; NTG, nitroglycerin; CSF, cerebrospinal fluid; ISF, interstitial fluid; MLV: Meningeal lymphatic vessels; GS, glymphatic system; PVS, perivascular space; EPVS, enlarged PVS; AQP-4, aquaporin-4: ROS, reactive oxygen species; TNF-α, tumor necrosis factor-α; CGRP, calcitonin gene-related peptide; IL, interleukin; DCLN, deep cervical lymph nodes.

In the pathophysiological network of migraine, the AQP4-dependent GS primarily mediates the exchange of CSF-ISF within the brain parenchyma, whereas MLVs regulate CSF efflux, drainage of CNS-derived antigens, and immune cell trafficking. CGRP may serve as a key molecular link between trigeminovascular activation and MLVs dysfunction, thereby connecting neuroimmune and vascular pathways. Furthermore, AQP4 plays a critical role in maintaining PVS function and modulating neuroinflammation: the accumulation of ROS and inflammatory cytokines triggers astrocyte hypertrophy and proliferation, leading to the release of additional inflammatory mediators and establishing a vicious cycle. These mediators compromise the structural and functional integrity of the PVS and GS. CSD induces transient PVS collapse and GS dysfunction, promoting a robust release of CGRP. Although CSF composition typically normalizes after CSD resolution, the inflammatory mediators released during CSD may sustain migraine pathogenesis. Neuroimmune interactions, neuroinflammation, and trigeminovascular activation are interwoven to form a complex regulatory network (**Figure 3**).
